# GLNet: global-local feature network for wheat leaf disease image classification

**DOI:** 10.3389/fpls.2024.1471705

**Published:** 2024-12-20

**Authors:** Shangze Li, Shen Liu, Mingyu Ji, Yuhao Cao, Bai Yun

**Affiliations:** ^1^ Aulin College, Northeast Forestry University, Harbin, China; ^2^ College of Computer and Control Engineering, Northeast Forestry University, Harbin, China; ^3^ School of Computer Science and Technology, Wuhan University of Science and Technology, Wuhan, Hubei, China; ^4^ College of International Studies, National University of Defense Technology, Nanjing, China; ^5^ Basic Education College, National University of Defense Technology, Changsha, China

**Keywords:** convolutional neural network, wheat leaf disease, image classification, multiscale features, GLNet model

## Abstract

Addressing the issues with insufficient multi-scale feature perception and incomplete understanding of global information in traditional convolutional neural networks for image classification of wheat leaf disease, this paper proposes a global local feature network, i.e. GLNet, which adopts a unique global-local convolutional neural network architecture, realizes the comprehensive capturing of multi-scale features in an image by processing the global feature block and local feature block in parallel and integrating the information of both of them with the help of a feature fusion block. By processing global and local feature blocks in parallel and integrating the information of both effectively with the help of feature fusion blocks, the model realizes the comprehensive capture of multi-scale features in images. This innovative design significantly enhances the model ability to understand the features of wheat leaf disease images, and thus demonstrates excellent performance and accuracy in the task of classifying wheat leaf disease images in real-world scenarios. The successful application of GLNet provides new ideas and effective tools for solving complex image classification problems.

## Introduction

Wheat is among the most extensively cultivated food crops worldwide, supplying vital sustenance and nutrition to billions of people globally (Erenstein et al., 2022). As reported by the Food and Agriculture Organization of the United Nations, wheat constitutes over one-third of the world’s cereal production and is a crucial component of the human food supply (Dadrasi et al., 2023). However, wheat is subject to a variety of diseases during its growth, including wheat leaf rust, powdery mildew, red mold and stripe rust (Singh et al., 2023). These diseases substantially diminish both the yield and quality of wheat, leading to significant losses in the agricultural economy (Rebouh et al., 2023). For instance, in the case of wheat blast disease, this condition not only impacts yield but also results in elevated levels of toxins in the grains, thereby posing a serious threat to both human and animal health.

Hence, timely and accurate diagnosis and management of wheat diseases are crucial for ensuring food security and sustainable agricultural development (John et al., 2023). Traditional methods of wheat disease diagnosis rely on field observation and empirical judgment of agronomists, which is not only time-consuming and laborious, but also involves a certain degree of subjectivity and risk of misjudgment. In modern large-scale agricultural production, there is an urgent need for an efficient and reliable automated disease diagnostic tool to improve the diagnostic efficiency and accuracy, to realize early warning and precise prevention and control (Edan et al., 2023).

Traditional methods of wheat disease diagnosis mainly include visual inspection and laboratory testing. The visual inspection method relies on the experience and knowledge of agricultural experts, and although it can be carried out in the field in real time, it is inefficient and highly dependent on experts (Guerrero et al., 2013). In addition, visual inspection is difficult to cover and detect quickly when dealing with large planting environments and is prone to missing early disease symptoms. Traditional laboratory testing methods, while accurate, are hindered by their cumbersome, time-consuming, and costly nature, making them impractical for large-scale monitoring and real-time diagnosis. In response to these challenges, automated and intelligent methods for diagnosing wheat diseases have emerged as key areas of research focus. Recent advancements in information technology and agricultural techniques have fostered the adoption of image processing technology for identifying and classifying crop diseases, marking it as a burgeoning diagnostic tool. However, traditional image processing methods rely on artificially designed features, which are difficult to adapt to the complex and changing field environment and diverse disease symptoms, and the classification effect is limited.

Deep learning, a leading technology in artificial intelligence, excels particularly in tasks involving image recognition and classification (Khasim et al., 2024). Convolutional Neural Network, as an important model of deep learning, mimics the processing of the human brain’s visual system through a hierarchical architecture, and is able to automatically extract multi-level features from image data, thus realizing efficient image classification and recognition (Ketkar and Moolayil, 2021). Deep learning methods have already achieved remarkable results in the fields of medical image analysis, automatic driving, security monitoring, etc., and have brought new hope for agricultural image processing. In the field of agriculture, image analysis techniques based on deep learning are gradually applied to crop pest detection and classification. By constructing large-scale image datasets and training deep learning models, automated and intelligent diagnosis of crop diseases can be realized. Deep learning methods exhibit superior accuracy and robustness compared to traditional methods, adeptly adapting to complex and dynamic field environments.

There have been some research attempts to apply deep learning to wheat disease image classification. Studies (Lin et al., 2019) have shown that deep learning-based methods for wheat disease classification have achieved better results to some extent. For example, some studies have used convolutional neural networks to classify wheat leaf diseases and achieved high classification accuracy. However, these methods based on convolutional neural networks often neglect the extraction of global features from wheat leaf disease images. Global features play an important role in wheat leaf disease images because they not only contain information about the overall distribution and morphology of the disease, but also capture important information about the environmental background, lighting conditions, and overall leaf morphology.

Therefore, to address this issue, we propose the Global Local Feature Network (GLNet) for classifying wheat leaf disease images. GLNet initially employs a bottleneck block, comprised of small convolutional kernels, to extract features from wheat leaf disease images. Subsequently, an inverted bottleneck block utilizes a large convolutional kernel to capture global features from the images, while another inverted bottleneck block, employing a similar architecture but with small convolutional kernels, extracts local features. Furthermore, a feature fusion block effectively enhances the interaction between these global and local features. The main contributions of this paper include:

1. a convolutional neural network-based wheat leaf disease image classification network, namely GLNet, is designed and implemented. GLNet improves its global feature sensing ability by introducing a large kernel convolution because of traditional convolutional neural network, which improves the accuracy of classification.
2. an effective feature fusion block is designed, which can effectively enhance the interaction between global and local features.
3. the effectiveness of the model is verified through experiments on a public dataset, and compared and analyzed with existing methods, demonstrating its potential in practical applications and proving the advantages of GLNet in image classification of wheat leaf disease.

## Related work

### Convolutional neural networks for image classification

Alexnet (Krizhevsky et al., 2012) with its unique network architecture and technological innovations such as ReLU activation function, Dropout regularization, data augmentation, and local response normalization, which opens up new paths in the field of deep learning. At the same time, by introducing deeper network layers, it is able to extract more levels of abstract features compared to the previous shallow networks, and thus performs well in dealing with complex image recognition tasks. The core design idea of VGGNet (Simonyan and Zisserman, 2014) is relatively simple and intuitive, which reduces the number of parameters of the model while increasing the depth of the network by connecting multiple smaller-sized convolutional kernels (usually 3x3) in series instead of larger-sized convolutional kernels. This design strategy not only improves the accuracy of the model, but also enhances the model’s ability to extract image features. ResNet (He et al., 2016) addresses the issues of gradient vanishing and model degradation in deep neural networks during training by incorporating residual connections. This innovation enables the successful training of deeper neural networks compared to previous models, resulting in significant improvements in performance across various tasks. InceptionNet (Szegedy et al., 2015) enhances model accuracy and performance through the introduction of the Inception block. This block facilitates simultaneous utilization of convolutional kernels of varying sizes and pooling operations at the same network level, enabling the capture of image features across multiple scales. By reducing computational demands and parameter count, this network architecture efficiently extracts features while managing resource consumption. It finds extensive application in computer vision tasks such as image classification and target detection. ACNet (Ding et al., 2019) improves model accuracy and efficiency by introducing asymmetric convolutional strategies. These enhancements strengthen feature extraction capabilities during training while maintaining consistent computation through convolution kernel fusion during inference. The fundamental concept involves allowing convolution kernels to dynamically adjust their size or shape based on input data, or combining kernels of different sizes to capture multi-scale features. This approach enhances the model’s understanding and generalization of complex scenes. EfficientNet (Tan and Le, 2019) achieves a unified scaling of the network depth, width and resolution through composite scaling techniques, thus significantly reducing the number of parameters and computation of the model while maintaining high performance. MobileNet (Howard et al., 2017) is a compact convolutional neural network architecture developed by the Google team. Its primary objective is to substantially reduce model size and computational complexity while preserving model accuracy. This design makes it particularly well-suited for deployment on mobile devices and embedded systems. DenseNet (Huang et al., 2017) greatly facilitates feature reuse and gradient propagation by using the output of each layer directly as the input of all subsequent layers through a dense connectivity mechanism, effectively mitigating the problem of gradient vanishing and improving the training efficiency and performance of the model. Meanwhile, DenseNet further reduces the number of parameters and improves the computational efficiency of the model by introducing Bottleneck and Transition layers to control the width and depth of the network. ShuffleNet (Zhang et al., 2018) effectively reduces the number of parameters and computational complexity of the model by adopting innovative techniques such as Group Convolution and Channel Shuffle.

### Image classification network for wheat leaf disease

M-bCNN (Lin et al., 2019) uses a unique convolutional kernel matrix arrangement, employing parallel convolutional layers. Techniques like DropConnect, exponential linear units, and local response normalization are integrated to combat overfitting and gradient vanishing. Compared to traditional networks, M-bCNN effectively boosts data streams, neurons, and connectivity channels with a modest parameter increase, enhancing its nonlinear mapping capabilities and data characterization. Feng et al (Xiao et al., 2021). constructed a wheat leaf disease image recognition model based on MobileNetV2 and used the training parameters on the ImageNet dataset as the initial parameters of the model. Jiang et al (Jiang et al., 2021). enhanced the VGG16 model through multi-task learning, leveraging pre-trained weights from ImageNet for transfer learning and fine-tuning to improve wheat leaf disease understanding. RFE-CNN (Xu et al., 2023) combines RCAB, FB, EML, and CNN to enhance Convolutional Neural Networks’ accuracy in classifying wheat leaf disease images.WR-EL (Pan et al., 2022) integrates multiple CNN models using bagging, snapshot ensembling, and SGDR algorithms to boost accuracy in wheat leaf disease image classification. Khan et al (Khan et al., 2022). developed an efficient machine learning framework for identifying and categorizing various wheat diseases, focusing on brown rust and yellow rust. The method involves several stages: initially, gathering data from diverse fields in Pakistan while accounting for illumination and orientation parameters. Next, preprocessing the data using segmentation and scaling techniques to distinguish healthy from affected areas. Lastly, training the machine learning model on the prepared dataset.Abdulaziz Alharbi et al (Alharbi et al., 2023). proposed a wheat disease classification network using Few-Shot Learning with EfficientNet as the backbone, capable of classifying 18 wheat diseases. Introduced an attention mechanism to enhance feature selection effectiveness. Bansal et al (Bansal et al., 2023). proposed a hybrid model for detecting and classifying wheat leaf spot diseases, combining Faster R-CNN for regional convolutional neural network-based detection with SVM for classification. Shafi et al (Shafi et al., 2023). Utilized a pre-trained U2 Net model for background removal and extraction of rust-affected wheat leaves. Applied deep learning classifiers, specifically Xception and ResNet-50, to assess the severity of stripe rust disease. Kukreja et al (Kukreja and Kumar, 2021). proposed a deep learning based method called Deep Convolutional Neural Network (DCNN) to automatically classify wheat rust infestation without human intervention. In addition, this DCNN training and testing process produced definitive and high classification results for wheat rust disease.

## GLNet

**Figure 1:**
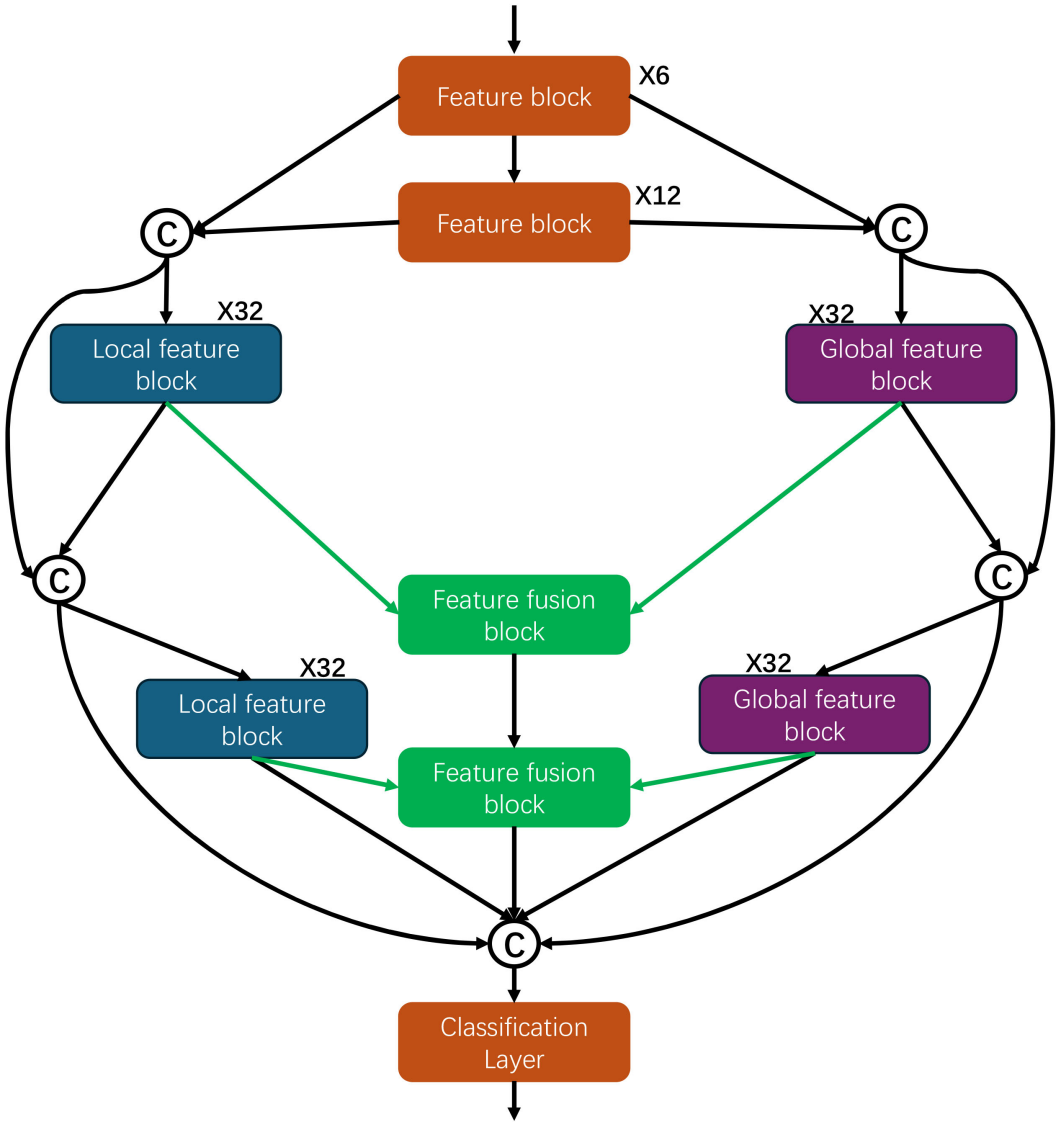
The GLNet architecture.

As shown in the **Figure 1**, GLNet mainly consists of the following parts, which are feature extraction block, local feature block, global feature block and multi-scale feature fusion block.

### Feature extraction block

**Figure 2:**
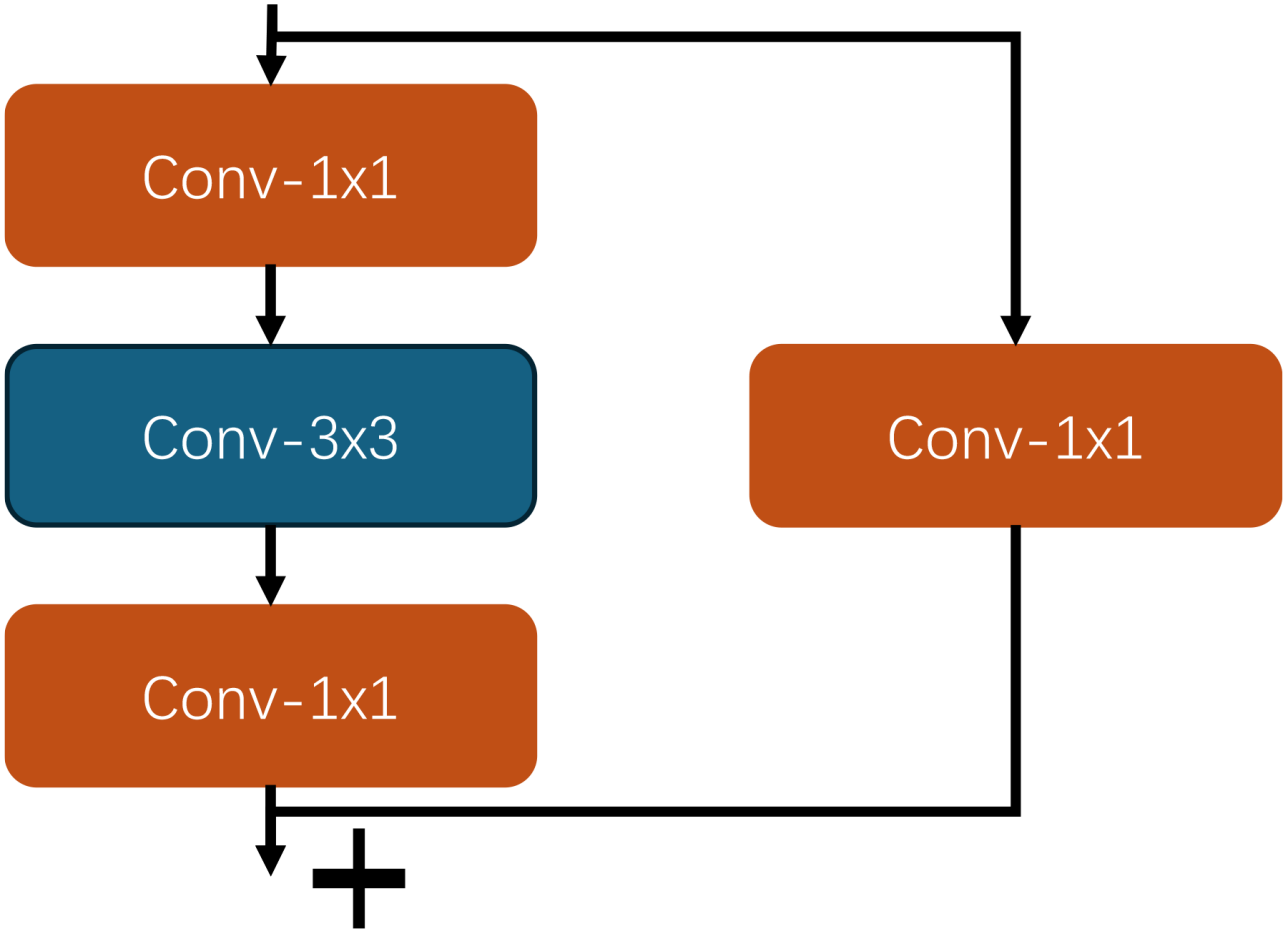
The feature extraction block architecture.

As shown in the **Figure 2**, the feature extraction block consists of two branches, the first branch consists of two 1x1 convolutional layers and a 3x3 convolutional layer. Specifically, the first 1x1 convolutional layer is used to reduce the dimensionality of the input features, thus reducing the computational complexity and the number of parameters. The next 3x3 convolutional layer is used to increase the sensory field, thus capturing more complex spatial features. Finally, a second 1x1 convolutional layer further extracts and combines the features.

The second branch is relatively simple and consists of a 1x1 convolutional layer. This 1x1 convolutional layer is mainly used to directly extract and combine input features, providing additional nonlinear transformations.

With the combination of these two branches, the feature extraction block is able to efficiently extract multi-scale and multi-level features, thus improving the expressiveness and performance of the model.

### Local feature block

**Figure 3:**
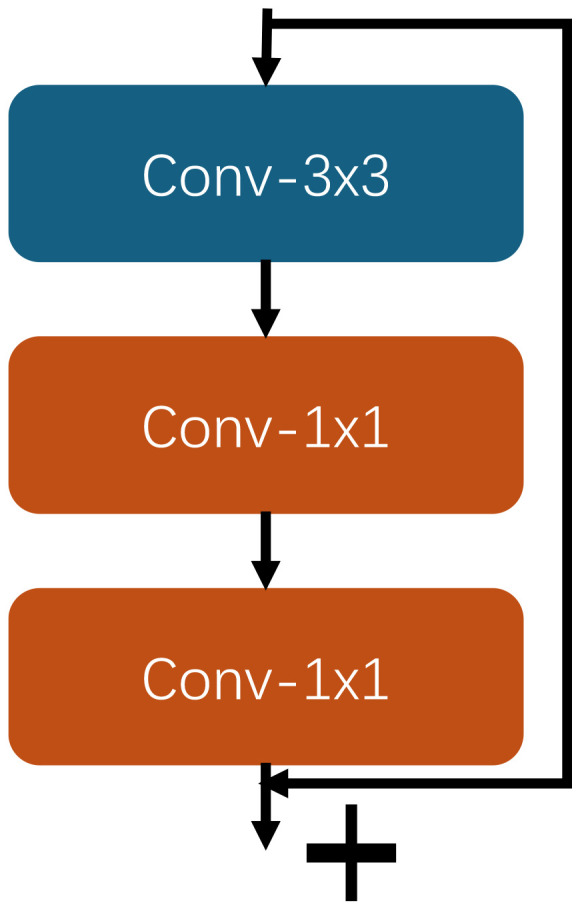
The local feature block architecture.

As shown in the **Figure 3**, the local feature block consists of two branches. The first branch consists of a 3x3 convolutional layer and two 1x1 convolutional layers. First, the 3x3 convolutional layer enhances feature representation by extending the receptive field to capture more complex and diverse spatial features. Following this, a 1x1 convolutional layer reduces input feature channel numbers, thereby decreasing computational complexity and parameters. Subsequently, another 1x1 convolutional layer further extracts and recombines features based on this dimensionality reduction.

The second branch is a Residual Connection that passes the input features directly to the output, skipping the intermediate convolutional operations. This connection helps to alleviate the gradient vanishing problem and promotes the training stability and efficiency of deep neural networks.

Through the combination of these two branches, the local feature block can effectively extract multi-scale and multi-level features, while the residual connection is utilized to maintain the stability and efficiency of the model training, ensuring that the input features are combined with the convolutionally processed features, thus achieving better feature learning results.

### Global feature block

**Figure 4:**
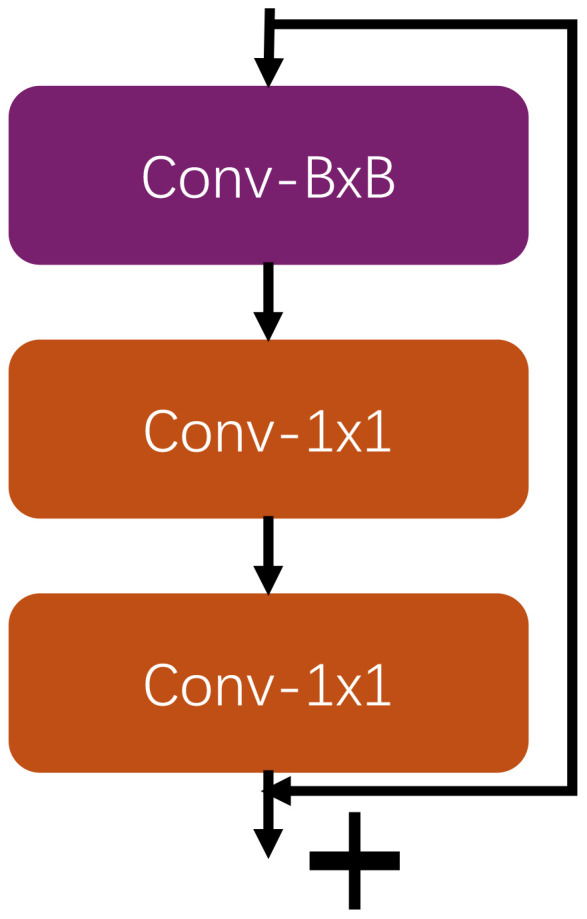
The global feature block architecture.

As shown in the **Figure 4**, the global feature block consists of two branches. The first branch consists of a convolutional layer sufficient to cover the size of the feature map and two 1x1 convolutional layers. First, the convolutional layer that is sufficient to cover the size of the feature map is used for global information extraction; this convolutional layer captures the global features of the entire feature map and provides richer contextual information. If the feature map size is 32, the convolution kernel size for Conv-BxB is 31. If the feature map size is 16, the convolution kernel size for Conv-BxB is 15. Then, the first 1x1 convolutional layer is used for dimensionality reduction to reduce the number of feature channels, thus reducing the computational complexity and the number of parameters. Next, the second 1x1 convolutional layer further extracts features and recombines them on the basis of dimensionality reduction to enhance the feature representation.

The second branch: is a Residual Connection, which passes the input features directly to the output, skipping the intermediate convolution operation. This connection can alleviate the problem of gradient vanishing and promote the stability and efficiency of deep neural network training.

Through the combination of these two branches, the global feature block can effectively extract global features, while using residual connection to maintain the stability and efficiency of model training, ensuring the combination of input features and convolutionally processed features, thus achieving better feature learning results. This design not only captures the global information, but also reduces the computational complexity through the 1x1 convolutional layer, making the model more computationally efficient while retaining efficient feature representation.

### Feature fusion block

**Figure 5:**
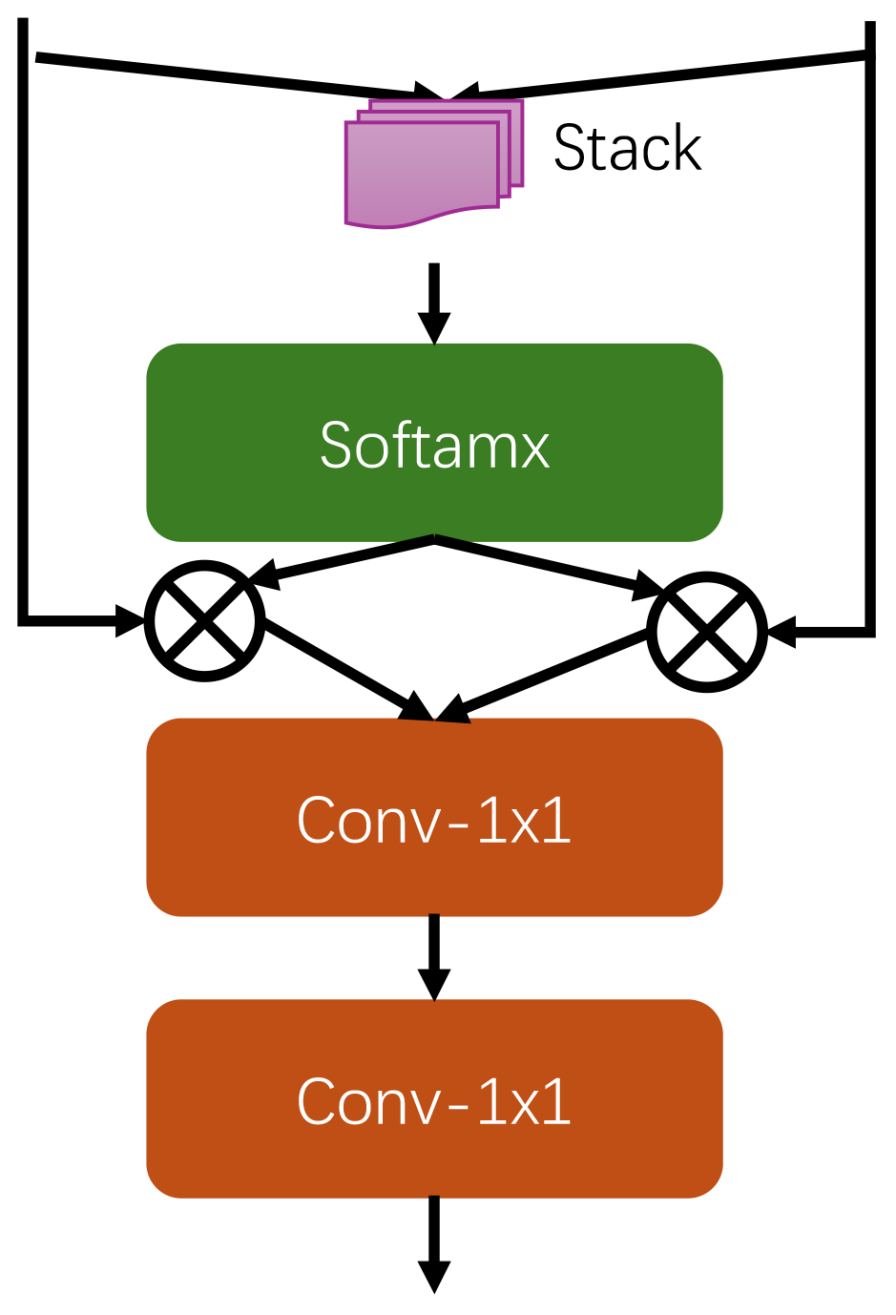
The feature fusion block architecture.

As shown in the **Figure 5**, feature fusion block mainly consists of two 1x1 convolutional layers and Softmax function. The process is as follows, first, the global and local features are stacked together to form a comprehensive feature map. Then, the Softmax function is used to calculate the weights of the global and local features so as to assign appropriate weight values to the features of both scales. The computed weights are then assigned to the original global and local features, thereby adjusting the importance of the respective features. The weighted global and local features are then summed element by element to form the fused features. Finally, the fused features are further processed to further extract and combine features through two 1x1 convolutional layers to enhance feature expression.

Through this process of feature fusion block, the global and local features can be effectively combined to make full use of multi-scale information, thus enhancing the feature learning ability and expression ability of the model. After the weight adjustment and element-by-element summing operation, the global and local features can work better together in the fusion process, and finally the feature representation is further optimized by the 1x1 convolutional layer to enhance the overall performance of the model.

### Classification layer

The classification layer consists of two fully connected layers, the first fully connected layer has an output dimension of 256 and is used to map the input features to a more compact feature space, thus capturing more discriminative features. The output dimension of the second fully-connected layer is the number of categories, which is responsible for mapping the features extracted from the previous layer to specific classification results. Each output node corresponds to a category, and the output values of these nodes are transformed into the probability of each category through the Softmax function. Magnetic tiles, a Dropout layer with a dropout rate of 0.5 is included between the two fully connected layers to prevent overfitting.The Dropout layer randomly discards half of the neurons, thus making the model more robust during training and avoiding over-reliance on training data.

Through this design, the classification layer can effectively extract and utilize the input features, and improve the generalization ability of the model through the Dropout layer, and finally achieve accurate classification results.

## Experiments

### Implementation details

GLNet is implemented based on Tensorflow and Keras with a batch_szie size of 40, epoch of 100, optimizer of Adamax, learning rate of 1e-4, and loss function of cross-entropy loss function. This paper reproduces all the comparison networks based on the same hyperparameters, and all the experiments in this paper are performed in a Tesla P100. The training is stopped when the accuracy does not increase for more than three epochs.

To evaluate the performance of GLNet and comparison networks, we use Accuracy (ACC), Precision (Prec), Recall and F1 score (F1). And the categories of Prec, Recall and F1 are balanced in a way using macro.

### Dataset

**Figure 6:**
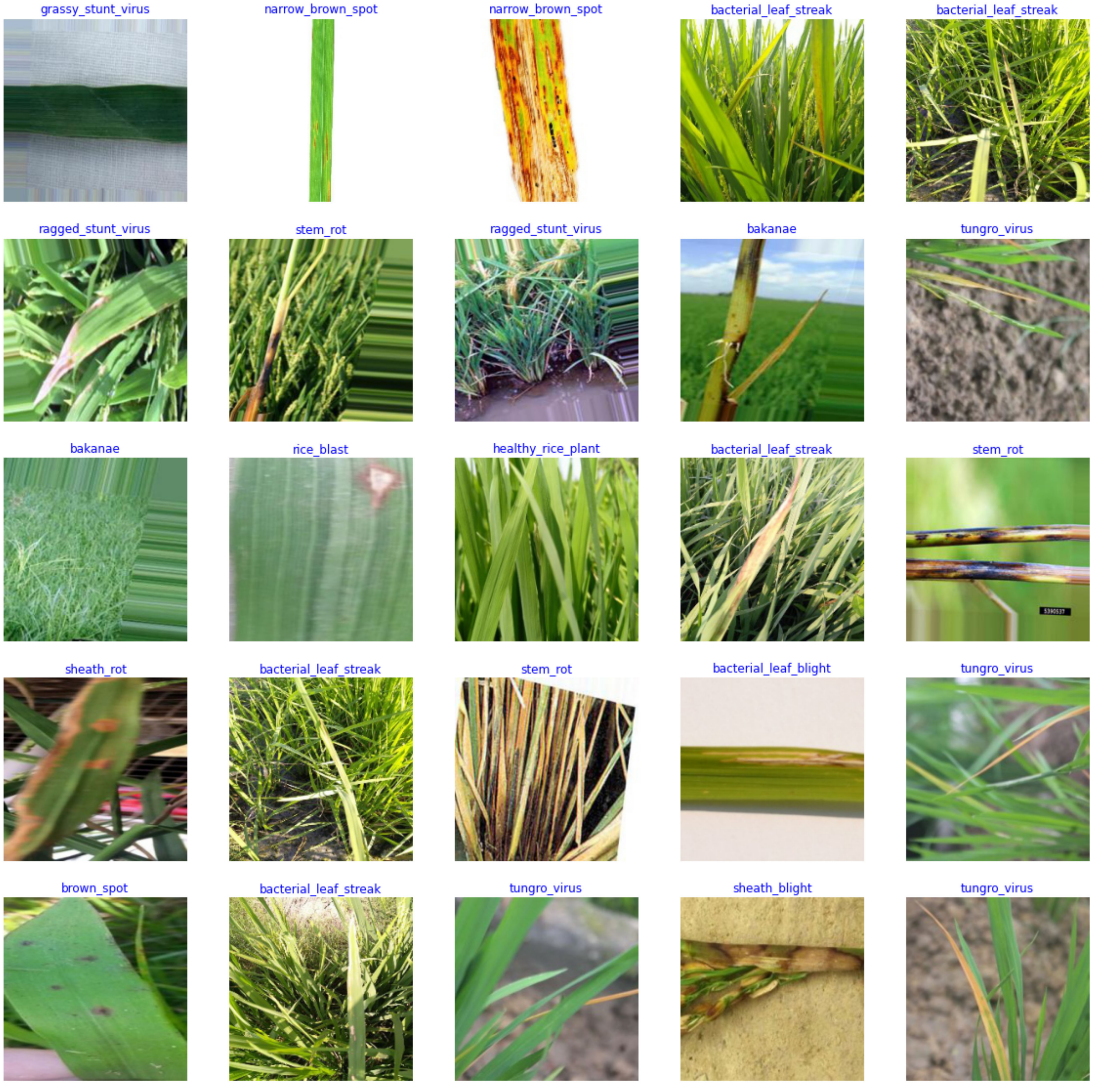
Example dataset.

This paper validates the performance of GLNet using the Philippines Rice Diseases dataset, which has a total of 14 categories. They are Rice Blast (140 photos), Sheath Blight (98 photos), Brown Spot (150 photos), Narrow Brown Spot (98 photos), Sheath Rot (98 photos), Stem Rot (100 photos), Bakanae (100 photos), Rice False Smut (99 photos), Bacterial Leaf Blight (140 photos), Bacterial Leaf Streak (99 photos), Tungro Virus (100 photos), Ragged Stunt Virus (100 photos), and Grassy Stunt Virus (100 photos). **Figure 6** shows examples of the different categories.

### Comparison experiment

To validate the performance of GLNet, we compare it with typical image classification networks including VGGNet, InceptionNet, InceptionNet, DenseNet, and EfficientNetb0.

We can get the following conclusions from the data in **Table 1**.

**Table 1:** Results of comparative experiments.

Network	Metric			
	ACC	Prec	Recall	F1
DenseNet121	0.9203	0.9258	0.9206	0.9197
DenseNet 169	0.9130	0.9156	0.9135	0.9115
EfficientNetB0	0.6594	0.7123	0.6611	0.6367
InceptionNet	0.9203	0.9232	0.9206	0.9202
ResNet156	0.7391	0.7714	0.7389	0.7384
ResNet 50	0.8696	0.8752	0.8706	0.8682
ResNet 101	0.7319	0.7464	0.7325	0.7321
VGGNet16	0.1159	0.1062	0.1143	0.067
RFE-CNN	0.8116	0.8616	0.8143	0.8076
DCNN	0.8623	0.8677	0.8619	0.8616
M-bCNN	0.8188	0.8336	0.8190	0.8202
GLNet	0.9638	0.9665	0.9635	0.9637

First, upon examining these results in detail, it becomes evident that traditional convolutional neural networks (CNNs) such as VGGNet16, ResNet152, ResNet50, and ResNet101, tend to excel at capturing local details but often overlook global contextual information, particularly in the context of wheat leaf disease image classification where this issue is particularly pronounced. Their performance, as indicated by metrics like ACC, Prec, recall, and F1 score, is generally lower compared to more advanced networks.

GLNet, on the other hand, addresses this limitation by introducing a global feature block that effectively captures the overall image architecture and contextual information, thereby compensating for the traditional network’s shortcomings in global feature perception. This enhancement allows GLNet to excel in understanding and classifying wheat leaf disease images, as evidenced by its top-tier performance across all metrics, with an accuracy of 0.9638, a precision of 0.9665, a recall of 0.9635, and an F1 score of 0.9637.

Second, GLNet leverages a combination of local and global feature blocks, seamlessly integrating the information from both through a feature fusion block. The local feature blocks focus on capturing local details and texture features within the image, while the global feature blocks provide a broader context and overall architectural information. By utilizing soft weight assignment and element-wise summation, the feature fusion block ensures that the advantages of both local and global features are comprehensively utilized. This dual focus enables GLNet to analyze wheat leaf disease images from a more holistic perspective, significantly improving recognition accuracy and robustness across different disease types.

In comparison to other advanced networks like DenseNet121, DenseNet169, EfficientNetB0, InceptionNet, RFE-CNN, DCNN, and M-bCNN, GLNet demonstrates superior performance, with higher accuracy, precision, recall, and F1 scores. This highlights the effectiveness of GLNet’s architecture in capturing both local and global features, which is crucial for accurately classifying wheat leaf disease images.

In summary, the superiority of GLNet in the wheat leaf disease image classification task stems from its ability to effectively integrate local and global features and achieve a more comprehensive feature understanding and expression through the feature fusion block, which improves the classification performance and the practicality of the model.

**Figure 7:**
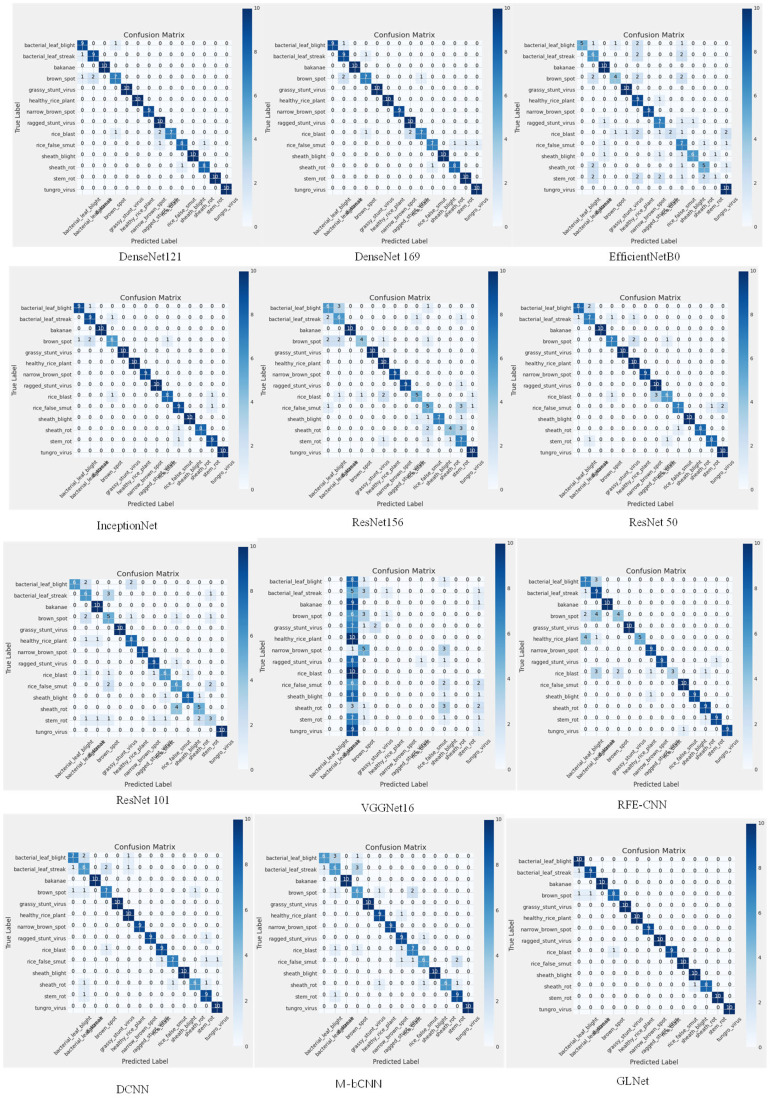
Comparison experiment confusion matrices.

As shown in detail in **Figure 7**, the GLNet model exhibits excellent classification ability for each category of wheat leaf disease images, and this remarkable result strongly demonstrates the effectiveness of the global feature introduction strategy. This strategy enables the model to capture and learn the complex features of wheat leaf disease images from a more comprehensive perspective, which greatly improves the classification accuracy and generalization ability.

### Ablation experiment

To verify the validity of different blocks in GLNet, we designed the following real: GLNet(w/o global) represents that GLNet does not use global feature blocks, GLNet(w/o local) represents that GLNet does not use local feature blocks, and GLNet(w/o fusion) represents that GLNet does not use feature fusion blocks.

By comparing the result of **Table 2**, we can get the following conclusions:

**Table 2:** Results of ablation experiments.

Network	Metric			
	ACC	Prec	Recall	F1
GLNet (w/o global)	0.9130	0.9149	0.9135	0.9115
GLNet (w/o local)	0.9493	0.9549	0.9492	0.9491
GLNet (w/o fusion)	0.9493	0.9541	0.9492	0.9497
GLNet	0.9638	0.9665	0.9635	0.9637

First, In the field of wheat leaf disease image recognition, local features play an important role. In the GLNet model, the local feature block is responsible for capturing these subtleties, which can be clearly demonstrated by the experimental results in **Table 2**. This is clearly evidenced by the experimental results in **Table 2**, where the ACC of GLNet (w/o local) is 0.9493, Prec is 0.9549, Recall is 0.9492, and F1 is 0.9491, which is a significant decline compared with the full GLNet. This indicates that in the absence of the local feature block, GLNet is difficult to effectively focus on detailed features such as the unique texture of localized lesions on leaves, and is unable to accurately differentiate and identify these key local lesion information, which in turn leads to a significant reduction in classification performance, highlighting the irreplaceable nature of localized features in providing precise detail information for the model to accurately identify different disease types.

Second, Global features are also indispensable in the task of wheat leaf disease image recognition, which is responsible for capturing the overall architecture of the entire leaf image as well as the background information. Leaf blade as a whole, its lesions are not only reflected in the local lesions, but also include the overall color change, the distribution of lesions on the leaf blade, and the contrast relationship with the surrounding healthy tissues, which are important clues reflecting the overall pathological state of the leaf blade. Analyzing the experimental data, the indexes of GLNet (w/o global) were relatively poor, with ACC of 0.9130, Prec of 0.9149, Recall of 0.9135, and F1 of 0.9115, which were much lower than that of the complete GLNet. Without the guidance of global features, GLNet will not be able to fully understand the overall pathology of the leaf, leading to inaccurate judgment of the overall lesion distribution and severity, thus affecting the improvement of classification performance.

The feature fusion block plays a key role in GLNet, which allows local and global features to work together. Wheat leaf disease images contain multiple levels of information from microscopic localized spots to macroscopic leaf overall status only when they are effectively integrated can they be maximized. For example, if local texture features are combined with global features such as overall color and spot distribution, the model will be able to judge and classify the disease more comprehensively and accurately. The data in **Table 2** show that the performance of the GLNet (w/o fusion) version shows a significant decrease, with ACC, Prec, Recall, and F1 of 0.9493, 0.9541, 0.9492, and 0.9497, respectively, which are different from the best performance of the full GLNet.

**Figure 8:**
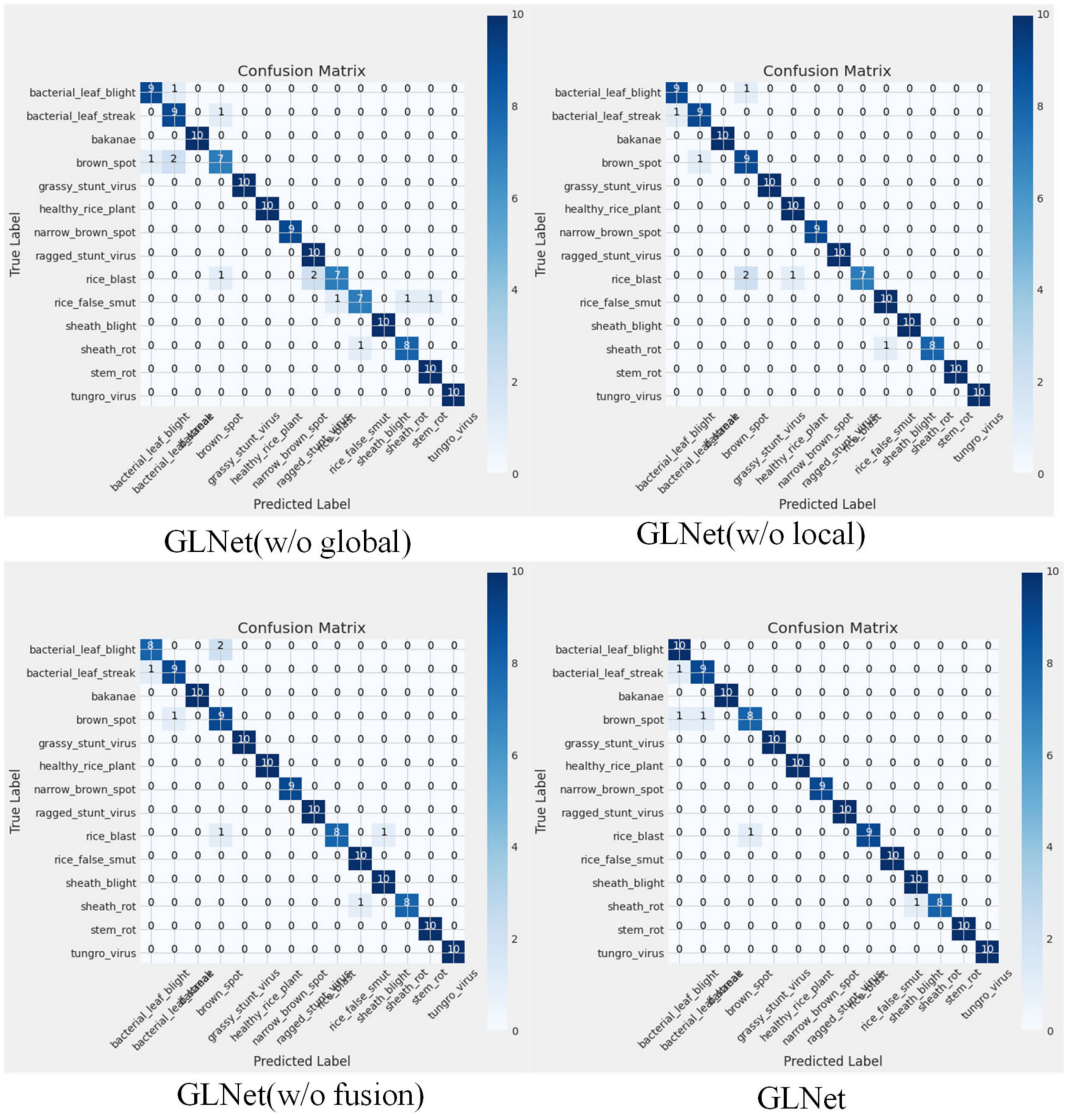
Ablation experiment confusion matrices.

As can be seen from **Figure 8**, we can clearly see that each building block in GLNet plays an active role in processing all types of wheat leaf disease images. It is worth noting that only when these blocks work in concert, i.e., are utilized simultaneously, GLNet can perform at its best and achieve optimal classification results. This fully illustrates the close cooperation and complementarity between the various components of the GLNet architecture, which together promote the overall model’s ability to recognize wheat leaf disease images.

**Figure 9:**
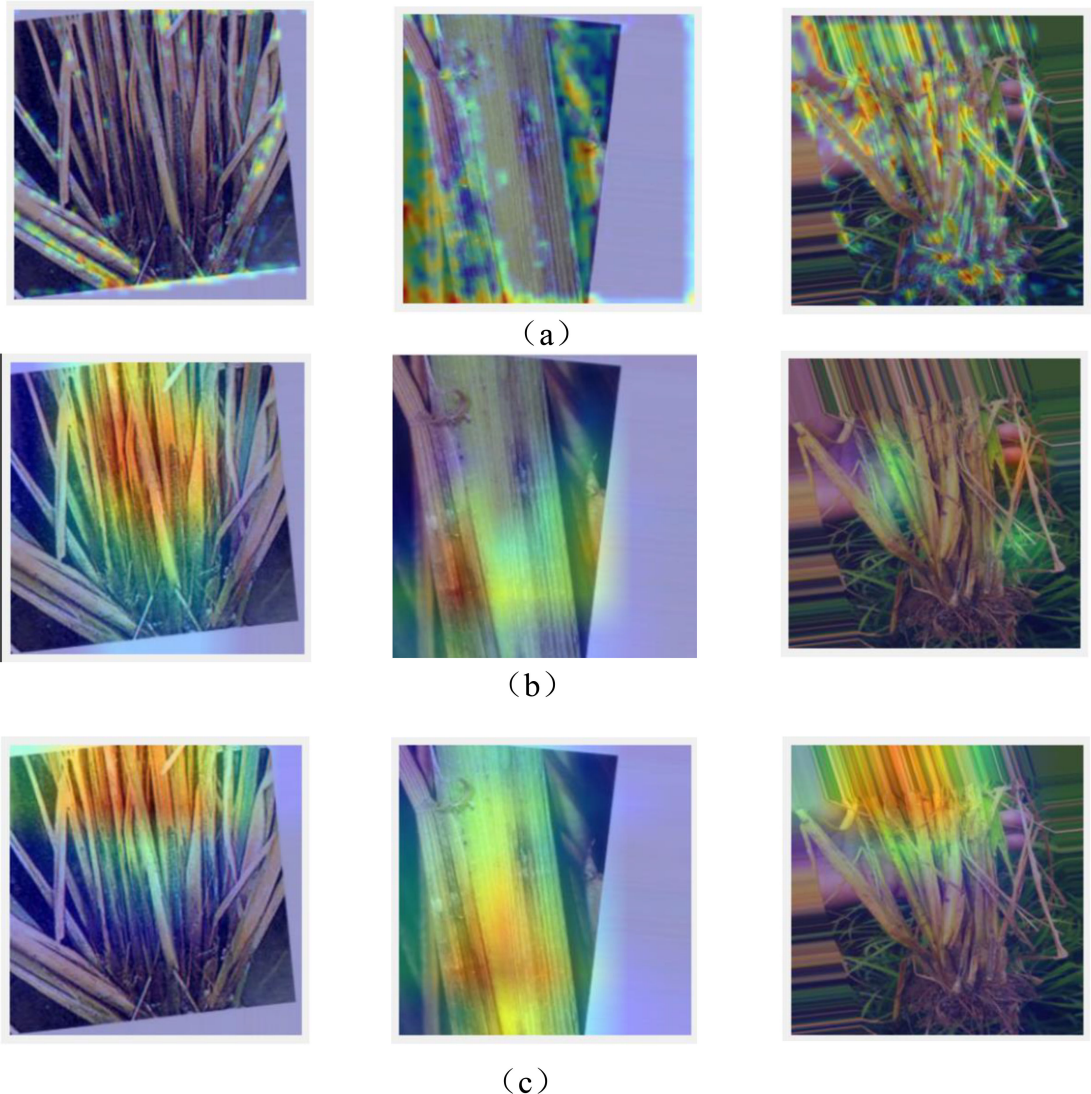
**(A)** Visualization of the input of the Local feature block and Global feature block. **(B)** Visualization of the output of the Local feature block. **(C)** Visualization of the output of the global feature block.

We visualized the output of Local feature block and Global feature block using Grad-CAM. As can be seen from **Figure 9**, we can see that the Local feature block can focus more on the local region of the wheat leaf disease image, while the Global feature block can focus on more regions than the Local feature block with its ability to learn global features.

## Conclusion

When dealing with the wheat leaf disease image classification task, traditional convolutional neural networks often face the problems of insufficient local feature perception and incomplete understanding of global information. To overcome these shortcomings, GLNet is proposed as a new solution in this paper.GLNet adopts a global-local network architecture, which effectively integrates local and global features by introducing parallel processing of global and local feature blocks and utilizing feature fusion blocks. This design not only enables the model to better capture the multi-scale features of an image, but also significantly improves the performance and accuracy in the wheat leaf disease classification task.

The innovation of GLNet is its ability to simultaneously process and fuse local details and global background information at different scales. Experimental results show that the performance of GLNet significantly decreases in the absence of local features, global features, or feature fusion blocks, further validating the effectiveness and necessity of its design. This makes GLNet a powerful tool for dealing with the task of classifying wheat leaf disease images and provides new technical support and methodology for disease identification and prediction in the agricultural field.

## Data Availability

The original contributions presented in the study are included in the article/Supplementary Material. Further inquiries can be directed to the corresponding author.
